# Differential Diagnosis and Treatment of Infantile Paralysis*Read as part of Symposium on Infantile Paralysis at the Medical Association of Central New York, Buffalo, October 19, 1916.

**Published:** 1916-12

**Authors:** Edward A. Sharp

**Affiliations:** Buffalo, N. Y.


### Differential Diagnosis and Treatment of Infantile Paralysis.*

By EDWARD A. SHARP, M. D., Buffalo, N. Y.

The diagnosis of Acute Epidemic Poliomyelitis presents a number of difficulties owing to the various clinical forms in which the disease appears.

Fully developed cases of the bulbo-spinal type offer no difficulty, and, while the majority of the cases in an epidemic usually conform to this type there are many obscure, irregular, atypical and abortive cases which are frequently unrecognized.

Lovett's definition that "Infantile paralysis is a general infection the results of which are most marked in the nervous system" indicates the indefinite and wide range of symptoms which may be expected in the atypical cases.

The early constitutional symptoms may differ in no respect from any ordinary febrile disturbance of gastro-intestinal or other origin. The fever, malaise, vomiting, constipation or occasionally diarrhoea, convulsions and general weakness are essentially those of any acute infectious disease, and, should the process stop at this stage, which it undoubtedly does in some cases, a diagnosis of infantile paralysis would be questionable even during an epidemic.

When, however, clinical manifestations of involvement of the nervous system are added to the above named symptoms, the diagnosis becomes more certain and if confirmed by the characteristic changes in the spinal fluid the diagnosis can be made without waiting for paralysis to complete the clinical picture.

Symptoms indicating an extension of the infection to the nervous system usually appear early—along with the prodromal constitutional symptoms, or after a day or two.

Hyperaesthesia is frequently present and the child cries when touched although at other times there is no pain and the child appears dopy.

Stiffness of the neck and back with tenderness on any attempt to raise the head or flex the spine is an early sign of meningeal involvement. The Kernig sign and the Brudzinski neck sign are usually present and the neck becomes retracted in the more severe cases. The increased pressure from fluid accumulation in the spinal canal and ventricles of the brain is shown by a tympanitic percussion note over the lateral ventricles—the so-called MacEwen's sign.

Twitching and jerking of the extremities or of muscle

---

*Read as part of Symposium on Infantile Paralysis at the Medical Association of Central New York, Buffalo, October 19, 1916.

groups, or a fine fibrillary tremor has been noted a number of times during the preparalytic stage. It is a symptom of spinal irritation, either from the toxins or from increased pressure.

A diminution of the reflex activity of an extremity may indicate that this part is to be paralyzed. Where observations could be made at frequent intervals the reflexes have shown a gradual diminution in activity up to complete loss. This is particularly striking in those cases where the corresponding extremity remains normal for comparison.

The mild cases, with little or no paralysis, are usually described as aborted and they may become a dangerous element in the spread of the disease.

Organisms have been described by Flexner, and more recently by Rosenow, one or the other of which may be the origin of the disease, but as yet no clinical-laboratory use has been made of the discovery to assist in diagnosis. The only positive laboratory method is the serum neutralization tests and the present technic is not practicable for routine work. The diagnosis must therefore be made on the clinical symptoms and the spinal fluid findings.

Examination of the spinal fluid may be of great value in determining the nature of an infection which shows an early involvement of the meninges such as occurs in poliomyelitis, tubercular meningitis, purulent meningitis, syphilitic meningitis, serous meningitis, etc.

During the past five months an investigation of fifty-six cases suspected of being poliomyelitis showed that in only fourteen cases was the diagnosis confirmed. Eleven of these occurred in the City of Buffalo, the remaining three were in Niagara County.

The atypical character of some of the cases made a diagnosis difficult without the aid of the spinal fluid findings. The fluid usually found in poliomyelitis, and which is characteristic of the infection, is clear or only rarely slightly opalescent. There is usually some increase in pressure and in our cases amounts varying from 2 c.c. to 20 c.c. have been withdrawn. Occasionally on standing over night the fluid has shown a fine fibrin-web formation but this is much less marked than in tubercular meningitis.

The cells, usually lymphocytes, are increased in amount from 20 to 300 or more per cubic millimeter. The large mononuclear cells are especially characteristic of the poliomyelitis fluid. There are no organisms found on staining.

The fluid also reduces Fehling's solution promptly and in no case has this reaction been absent.

The amount of albumen as tested with nitric acid, and of globulin tested with the butyric acid reaction of Noguchi, shows an increase.

Seven cases of tubercular meningitis were found during the investigation. In most of these the early clinical signs were indefinite and resembled the atypical poliomyelitis cases. The constitutional symptoms were more prolonged; the cerebral symptoms as twitchings, rigid attacks and strabismus were usually more marked. The fluid obtained showed some of the physical characters of the poliomyelitis fluid,—being clear, under increased pressure and with fibrin-web formation. The cell count is usually high, mostly lymphocytes, although in two instances the cells were fewer than the average poliomyelitis fluid; 60 in one case and 78 in another.

The reaction for albumen and globulin are usually more marked while the reduction of Fehling's solution is much less marked than in poliomyelitis. In several cases there was practically no reduction at once but after cooling a fine film of the copper deposit could be seen.

In four of the cases tubercle bacilli were found in the spinal fluid.

In contrast to these cases with clear fluid were six cases of purulent meningitis, three due to the meningococcus and three to the pneumococcus. The organisms are present in the purulent fluid and are identified by proper staining. The cells are practically all polymorphonuclear. Two of the cerebro-spinal meningitis cases due to meningococcus infection recovered under serum therapy. The three pneumococcus cases died.

One case of serous meningitis showed the typical symptoms of an acute meningeal infection such as occurs during the early stage of poliomyelitis, but the spinal fluid was negative except for the great increase in quantity. The cell count, albumen, globulin and Fehling's reaction were normal. Recovery followed promptly after removing over thirty cubic centimeters of spinal fluid.

During the course of acute infectious diseases as measles, influenza, typhoid fever, pneumonia, etc., meningeal symptoms may develop due to the action of the toxins without direct invasion of the meninges by the bacteria. This reaction is called meningismus or Dupre's Disease. Several cases were observed during the recent investigation. Two cases with measles developed a temporary weakness of the extremities lasting a day or two but recovered completely. Probably the toxins of these acute infections produce a temporary suppression of the anterior horn cells of the spinal cord similar to that which occurs in the epidemic form of poliomyelitis, or a type of peripheral neuritis develops such as occurs in diphtheria, alcoholism, etc.

One case of scurvy and several cases of rickets had pains in the joints and tenderness on manipulation producing loss of function which was thought to be poliomyelitis. The reflexes were normal and there was no actual muscle weakness. The characteristic nutritional changes and hemorrhagic areas confirmed the diagnosis of scurvy and of rickets.

Acute arthritis due to rheumatism or other infections has been observed several times and the loss of function of the extremity attributed to paralysis. There was no change in the reflexes and the loss of function was due entirely to pain and swelling of the joint.

Five cases of acute encephalitis with convulsions and temporary weakness of one side of the body from exhaustion or actual hemorrhage were observed. It is more difficult to classify the infection in these cases as a similar condition may result from the poliomyelitis infection,—the so-called polioencephalitis.

Two of these cases had severe gastro-intestinal disturbance with foul stools and considerable mucous. The fever was higher than usually occurs in poliomyelitis and the prodromal and meningeal symptoms were absent.

In one case with convulsions lasting several hours, followed by hemiplegia, the child died during the convulsive attack. Autopsy showed a marked congestion of the entire cortex with edema and a thickened, adherent dura which was not of recent origin. The dura was so strongly adherent to the brain and skull that the brain could not be removed without tearing. This was most marked over the right motor region. Adhesions were found along the fissure of Sylvius but no tubercles could be found. The spinal fluid obtained a few hours before death was clear and showed none of the characteristic reactions of poliomyelitis.

Treatment: Aside from the usual quarantine and isolation of the patient certain prophylactic measures would seem to be indicated. Considerable evidence has been adduced to show that the naso-pharynx is the usual portal of entry of the virus. A naso-pharynx free from excessive lymphoid tissue, as tonsils and adenoids, would be desirable as offering a less favorable field for infection. The repeated use of antiseptic nasal douches or applications should be discouraged as it is liable to lower the resistance of tissues which may be already susceptible. Applications of Argyrol 25% solution may be made in cases where exposure or contact has occurred.

One feature of an epidemic which has been constantly observed is the limited susceptibility of the majority of the population, and this fact must be taken into consideration in estimating the value of any prophylactic or quarantine measures. A similar conservatism must be placed on the value of any method of treatment as the acute infection is selflimited and is liable to stop at any stage of the disease.

The treatment at the onset is that of any febrile attack. One of the first indications is active catharsis and elimination. Calomel and salines or castor oil, followed by bowel washes should be given at the onset.

The child should be kept in bed and avoid any exertion of the weakened or paralyzed parts, and this rest should be enforced during the period of fever and hyperaesthesia. As long as the temperature remains high there is danger of an extension of the paralysis.

A careful regulation of the diet is necessary during the acute febrile stage, and a liquid diet is preferable for a few days.

Urotropin, which was so extensively used during the 1912 epidemic, has not been used except for a few days at the onset but it may be given as a prophylactic in exposed cases. After the infection has invaded the nerve structures its application is useless.

Adrenalin has produced some startling results in checking the paralysis where this is due to edema, but it has no influence on the direct action of the toxines on the anterior horn cells. Its use, however, may tide over an emergency by checking the edema and it should be given in doses of 2 c.c. of 1:1000 solution every six hours, injected intraspinally after withdrawal of a few cubic centimeters of spinal fluid.

The repeated withdrawal of the spinal fluid probably exerts a beneficial influence by removing some of the virus or toxins and this may account for some of the success attributed to the use of adrenalin. No unfavorable results have followed the repeated lumbar puncture nor the administration of adrenalin.

Adrenalin has been of most value in the treatment of the rapidly advancing cases of the Landry's paralysis type, and its use in conjunction with oxygen inhalations and artificial respiration has been successful in those cases of intercostal paralysis which appeared to be advancing toward a fatal termination.

The rational specific treatment of poliomyelitis would be a serum capable of neutralizing the infection before the nervous structures are injured and which could also be used as a prophylactic in immunizing those exposed or susceptible.

Up to the present time no artificial serum has been capable of meeting these requirements. It has been found, however, that the blood-serum of patients who have recovered from epidemic poliomyelitis contains a substance capable of neutralizing the virus of an active case, and that monkeys inoculated with a virus so treated do not develop the disease.

As a result of these experiments the blood serum of recovered cases has been employed by Netter, Flexner and others in the treatment of acute cases. In some instances the serum was obtained from patients who had recovered many years previously,—thirty years in one case reported by Netter.

The serum is prepared by withdrawing sufficient blood and separating the clear serum which may be kept in sealed ampoulles ready for use. 20 to 30 c.c. of this serum may be injected intraspinally every day or two for several treatments after removal of an equal amount of spinal fluid. The prevailing opinion is that the serum should be used very early, before any paralysis has developed, although some of the clinicians think that the early use tends to aggravate the symptoms, increasing the fever and producing a severe meningeal reaction.

During the acute stage the indications are to keep the child quiet and to avoid meddlesome mechanical therapeutics as massage, electricity, hydrotherapy, etc., although at a later stage, when the hyperasthesia and tenderness have disappeared, these methods may be used to advantage. A light bandage or fixation splint may be used during the acute period to relieve the pain and tenderness which would result from movement, and to prevent contractures and deformities which almost invariably occur when one muscle group is paralyzed while its antagonist remains normal.

After subsidence of the acute symptoms the treatment is orthopaedic and muscle training, with assistance of massage, hydrotherapy, and occasionally electricity.

---

Note: The third part of the Symposium on Poliomyelitis, dealt with the Surgical Treatment and was given by Dr. W. W. Plummer of Buffalo. As his remarks have already been embodied in other papers, he has decided not to publish them.

---

**Peripheral Neuritis Following Emetine.** A. R. Kilgore, Shanghai, Boston M. & S. Jour., Sept. 14, report 6 original cases and include 5 of Levy & Rowntree, in which neuritis of varying severity followed a total dosage og .25—1.25 of emetine for amoebic dysentery (4-19.5 grs.). They refer to the condition as fairly common. The prognosis is good but the symptoms may persist for several weeks.

---

**Caesarian Triplets.** The Boston M. & S. Jour., Oct. 5, reports a case delivered at Providence Hospital, Holyoke, Mass., Sept. 21. The babies were all vigorous and weighed 6 pounds each for two girls and 5 pounds 12 ounces for a boy.

